# Making it possible to measure knowledge, experience and intuition in diagnosing lung injury severity: a fuzzy logic vision based on the Murray score

**DOI:** 10.1186/1472-6947-10-70

**Published:** 2010-11-04

**Authors:** Carlos E D'Negri, Eduardo L De Vito

**Affiliations:** 1 Consejo Nacional de Investigaciones Científicas y Técnicas (CONICET), Combatientes de Malvinas 3150, CP 1427, Buenos Aires, Argentina; 2 Departamento de Neumonología y Laboratorio Pulmonar. Instituto de Investigaciones Médicas Alfredo Lanari, Universidad de Buenos Aires, Buenos Aires, Argentina

## Abstract

**Background:**

Murray score is the result of an equation that gives all its variables the same linear contribution and weight and makes use of consented cut-offs. Everyday physicians' vocabulary is full of terms (adjectives) like: little, small, low, high, etc. that they handle in an intuitive and not always linear way to make therapeutic decisions. The purpose of this paper is to develop a fuzzy logic (FL) vision of Murray's score variables to enable the measurement of physicians' knowledge, experience and intuition in diagnosing lung injury and test if they followed Murray's equation predictions.

**Methods:**

For a prospective survey carried out among a team of professionals (aged 29 to 53) in a University Hospital Intensive Care Unit, twelve physicians filled in two questionnaires. In the first one they had to define the ranks which should be categorized as normal, moderate and severe for three of four Murray variables. In another questionnaire, which represented all probable combinations of those categories, they had to tick the pulmonary condition as: no injury, mild, moderate, and ARDS. This procedure gave rise to a Fuzzy Inference System designed to provide the *degree of severity *as *sensed *by the group.

**Results:**

The survey showed fuzzy frontiers for the categories and fuzzy diagnosis. In all, 45% of the hypothetical patients (n 18,013) were equally diagnosed by the survey and Murray's equation, whereas another 51% was overestimated in one level by the survey. Physicians agreed with 96.5% of ARDS cases according to Murray's test but only 11.6% of its mild cases were equally diagnosed by the survey. Nonlinearity of the survey reasoning (high relevance to gas exchange and chest film) was apparent.

**Conclusions:**

The contiguous categories of the variables confirm the existence of fuzzy frontiers. An overestimation was found in the surveyed group's interpretation of severity. This overestimation was mainly due to the different weight assigned to PO_2_/FiO_2 _and chest film variables. The FL approach made it possible to measure knowledge, experience and intuition as they appear in physicians' thinking. FL methodology could overcome a series of restrictions that current tests have due to cut-offs

## Background

Physicians' everyday vocabulary is full of adjectives like: little, great, much, small, low, high, moderate, etc. which they handle in an intuitive way to make therapeutic decisions. These are concepts that cannot be measured, which operate in a physician's logic and lead to a precise output: a decision based on those subjective modifiers. It is just a matter of concept, perception and good criterion.

Clinical practice in intensive care units resorts to many severity scores. They are calculated by averaging available items which make use of cut-offs that force an abrupt change of categorization of a variable when it has only slightly moved across one of its frontiers. This is in clear opposition to the smooth transitions implicit in intuition.

Murray's score is the average of four variables proposed for an expanded definition of the acute respiratory distress syndrome (ARDS) to facilitate the study and treatment of acute lung injury [1]. It allowed researchers and clinicians to speak a common language when discussing degrees of hypoxemic respiratory failure. The American-European Consensus Conference Committee (AECCC) [2] later suggested the term acute lung injury (ALI) and defined ARDS as a more severe form of ALI. Murray's score gives all its variables the same weight and together with AECCC both make use of cut-offs. Other tests have been proposed [3,4]; however, the Murray score has reached recognition and is still considered very useful in defining the severity of ARDS in clinical and research studies.

To assess the way in which Murray's variables are evaluated by physicians at the time of diagnosing ALI and ARDS, we resorted to the fuzzy logic (FL) approach which allowed us to design a fuzzy inference system (FIS), which would represent the knowledge, experience and intuition of a group of physicians surveyed. FL is a generalization of classical logic [5,6]. The latter accepts only two possibilities for a proposition: to be true (assigned a 1 conventionally) or to be false (assigned a 0 conventionally). The former admits a continuum between 0 and 1. Things can be partly true and false at the same time. The Murray score runs across the whole range of severity, from no injury to ARDS, thus allowing us to compare any degree of severity at the time of testing FIS. FL deals with the above-mentioned adjectives of such fuzzy contours and, following the logic that connects them, it is possible to obtain an output or reach a conclusion. FL is a plausible attempt to approach human thinking (for a brief introduction to FL see Appendix I).

As far as we know, two studies [7,8] have proposed to apply the FL theory in order to determine the severity of ARDS, but there is a lack of information about ALI-ARDS scoring and its relationship with human intuition. This paper presents an FL vision of Murray's score variables, which makes it possible to measure knowledge, experience and intuition in diagnosing lung injury severity. Finally, FIS, and not the physicians, was inquired with hypothetical cases, covering a broad spectrum of data, to obtain its lung injury severity diagnoses as an output. Advance information of this work was presented at the 2009 ATS International Conference [9].

## Methods

We carried out a prospective observational study in which twelve physicians (aged 29 to 53) were surveyed. This group comprised the attendant staff of the intensive care unit at the *Instituto de Investigaciones Médicas Alfredo Lanari*, University of Buenos Aires, Argentina.

**Figure 1:**
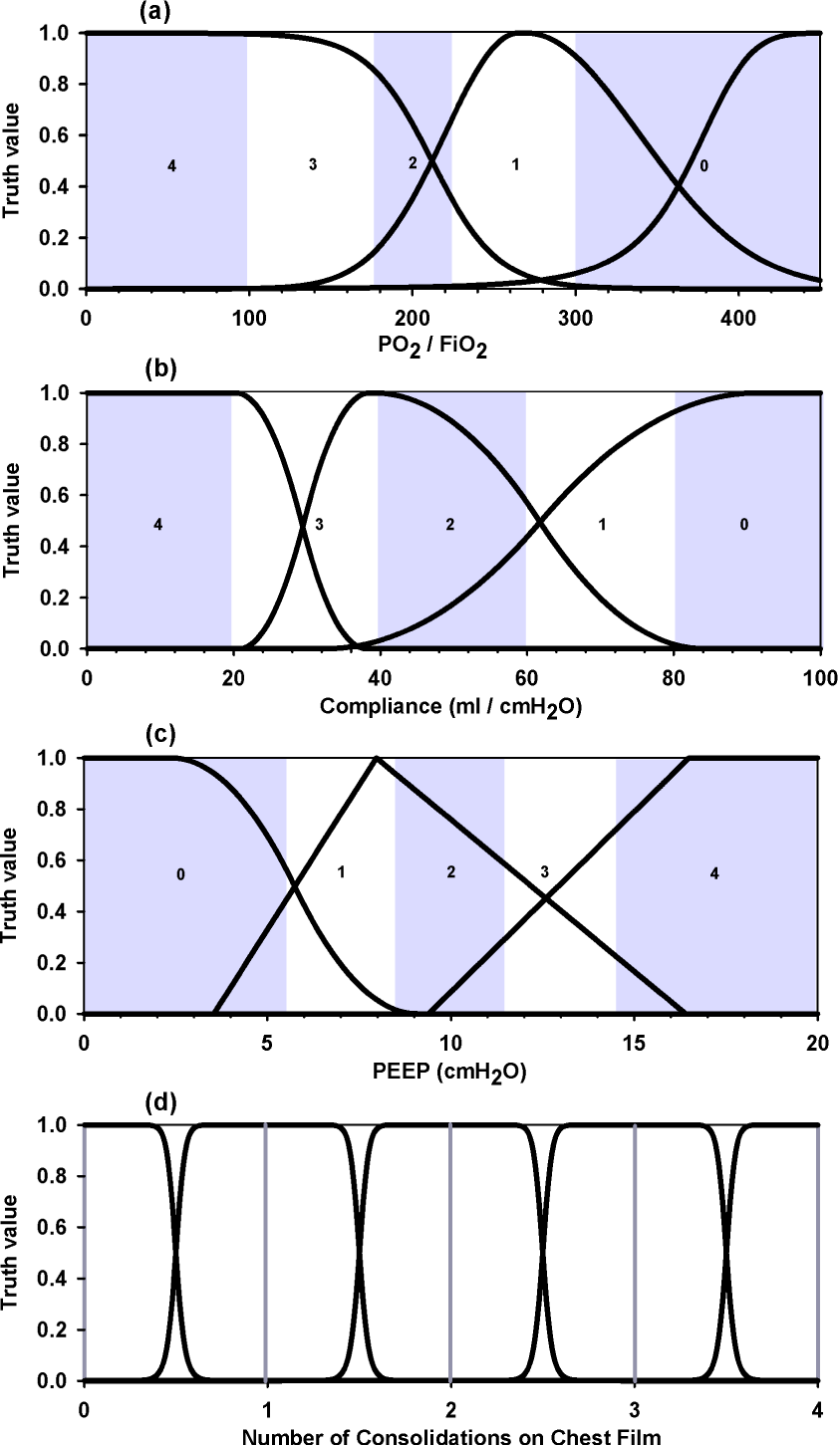
**Membership functions of Murray variables**. (a) PO_2_/FiO_2 _functions: severe, moderate and normal (left to right). (b) Compliance functions: severely diminished, moderately diminished and normal (left to right). (c) PEEP functions: low, moderate and high (left to right). (d) Chest film functions: for 0 through 4 quadrants consolidations; vertical lines at 0, 1, 2, 3 and 4 represent Murray's values assigned to this variable. Murray bands (grey and white strips) were drawn superimposed in the first three graphs and bands' values are seen embedded in them.

Physicians were requested to fill in a form that included two questionnaires. The first one was related to the following three variables: PaO_2_/FiO_2 _ratio (values from 0 to 450 were subdivided in 9 equal segments), Compliance (values from 0 to 100 ml/cmH_2_O were subdivided in 10 equal segments ) and PEEP (values from 0 to 20 cmH_2_O were subdivided in 10 equal segments) in which they had to indicate which range was to be assigned to each of a three-category division (low, medium, high) with no overlapping and no gaps in between. Of course, coincidence among all those surveyed proved to be impossible. This fact introduces the first aspect of fuzziness: individual criteria for the above-mentioned adjectives are not unique and thus fuzzy frontiers arise. With these variables it was possible to construct nine membership functions (figure 1a, b, c) [see procedure in Appendix II (a)]. The fourth variable (chest film) strictly followed the five categories defined by Murray's score (figure 1d), [see details in Appendix II (b)].

Once a mathematical description of the fuzzy concepts representing the categories of the variables was obtained, a body of rules involving these concepts was built; this corpus could show how a physician's intuition decides upon the severity degree of the hypothetical patient line in the second questionnaire of that form, where variables are not specific values but ranks instead. The second questionnaire showed all possible and probable combinations of those three categories plus the five possibilities of Murray's classification of chest film (107 rules). The physicians had to tick just one of the four following categories into which the pulmonary condition should fall: no injury (assigned a value of 0), mild (pulmonary injury without ALI, assigned a 1), moderate (pulmonary injury with ALI, assigned a 2) and ARDS (assigned a 3). The degree of severity will not be a set of membership functions as we do not have an "*a priori*" continuum of values addressing severity. That continuum would, in turn, be the by-product we are seeking. Instead, our interest was to define four possible states for the output, as if they were crisp numbers, to be able to compare them with the four Murray levels of injury that arise after splitting the 'mild to moderate' interval he proposed into two halves (through the 1.5 score) [see Appendix II (c) for attainment of FIS output].

The FIS obtained from the survey was fed with 18,013 quadruplets, representing the four variables -- the values of which spanned the whole range of each one [see Appendix II (d) for the choice of data procedure] - and provided us with the different severity degrees. The same quadruplets were introduced into the Murray equation to obtain the corresponding Murray score.

### Analysis

Analysis and data management were carried out using FL tools and programming environment of MATLAB Software, The MathWorks, Inc. The kappa coefficient was used to evaluate concordance between Murray's score and the opinion of the physicians surveyed.

## Results

After the survey was completed, membership functions for Murray's variables were obtained (figure 1). Overlapping of membership functions is apparent, contrasting dramatically with Murray's cut-off bands. These functions together with the completion of the second questionnaire, which also showed non-uniformity in diagnosing, determined the FIS alluded to under "Methods".

**Figure 2:**
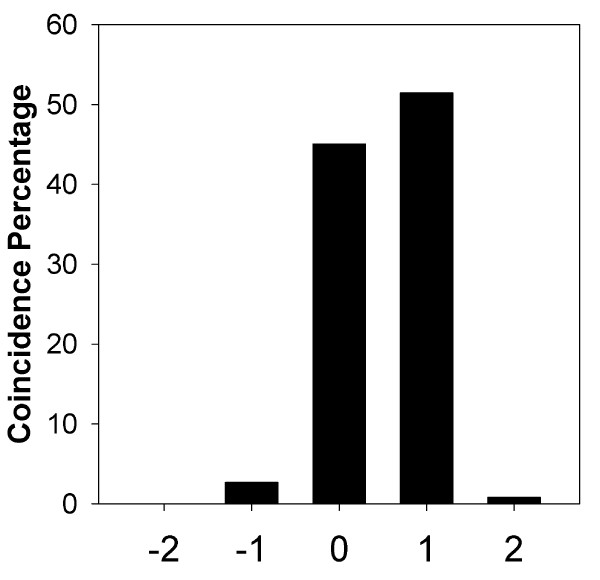
**Coincidence percentage between Murray and Survey**. Y axis: percentage of total population. X axis: 0 means total coincidence whatever the level of injury, +1 addresses for one more level of severity according to the survey, whatever the level established by Murray and +2 for two more levels by the physicians; -1 and -2 represent underestimation.

Figure 2 shows a histogram of the coincidence percentage between both methods [see Appendix II (e) for a description on the management of both methods' outputs]. It can be seen that agreement was obtained in 45% of all quadruplets; overestimation by one level, on the part of the survey, represented 51%, and by two levels it was less than 1%. Conversely, underestimation by one level represented less than 3% and by two levels less than 0.04%. The weighted mean of its abscissa values is 0.503 ± 0.567 (SD), 95% CI: 0.495 - 0.511, which indicates a significant skewness towards overestimation. By removing coincidence, the mean of disagreement was 0.915 ± 0.455 (SD), 95% CI: 0.907 - 0.925. According to this analysis there was a tendency by physicians to diagnose one more degree of severity than Murray.

Table 1 shows the detailed distribution of all cases according to severity degrees in terms of Murray and the Survey. A progressive agreement of diagnosis from mild to severe is apparent. As a whole, there was a slight agreement between Murray's score and that of the physicians surveyed [kappa coefficient: 0.1572 ± 0.0066 (SE), 95% CI: 0.1443 - 0.1701]. In turn, the comparison between severe degrees of lung injury (ARDS) and the rest of the conditions showed a moderate agreement [kappa coefficient: 0.4321 ± 0.006 (SE), 95% CI: 0.4204 - 0.4438].

**Table 1:** Number of cases - Distribution according to severity degree

	Murray severity degree			
	
	0	1	2	3
**Fuzzy severity degree**				
0	9	85		
1	18*	656	282	6
2		4776*	4139	114
3		145	4471*	3312

The numbers in row and column headings run from no injury (0) to ARDS (3).

* Fuzzy overestimation by one level.

The next step in the analysis was to explore the approach used by the physicians to ponder the different bands of all variables along the progression of diagnostic severity from no-injury to ARDS. The main cause of injury overestimation in the survey was the assignment of greater importance to low values of PaO_2_/FiO_2 _and high values of consolidations, which produced migration of cases from those regions towards one more degree of severity, [see Appendix II (f) for the analysis that arrives at these conclusions]. On the basis of this rationale, it was observed that cases with better values of Compliance and PEEP also migrated in the same direction.

### Fuzzification of Murray's equation

**Figure 3:**
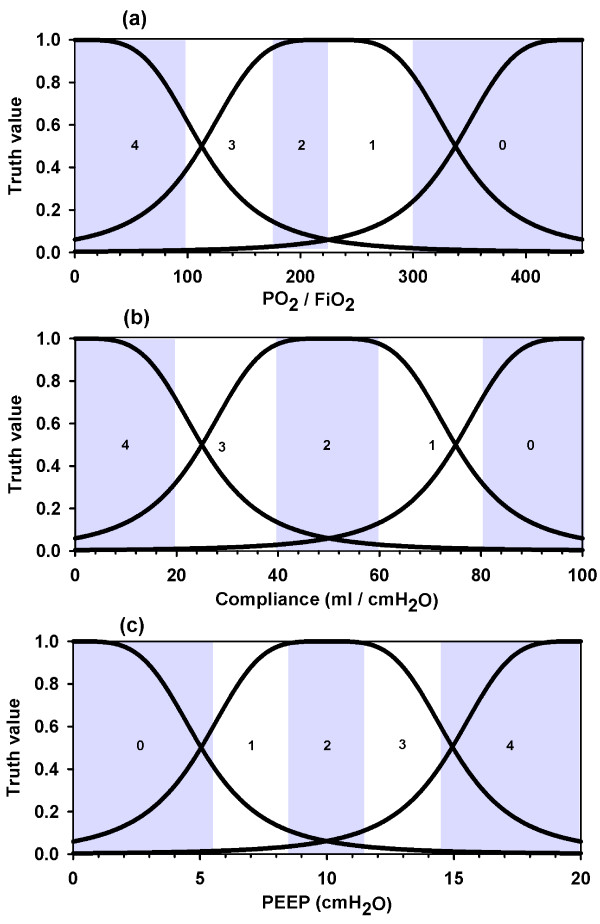
**Fuzzification of Murray's equation**. (a) PO_2_/FiO_2 _membership functions. (b) Compliance membership functions. (c) PEEP membership functions. These are the membership functions of an equivalent fuzzy inference system that responds as Murray test does, obtained through a neuro-fuzzy adaptative inference system imposing the same number of categories as the Survey fuzzy system. Murray's bands are shown as in figure 1.

Based on the Murray output for our input cases, we went all the way in the opposite direction and obtained its corresponding FIS by appealing to a *neuro-fuzzy adaptative inference system *conditioned to have the same number of categories as the survey's fuzzy system and with the scores predicted by Murray's score for the 18,013 quadruplets as the output. Figure 3 shows the remarkable symmetry of its membership functions. The reason must be sought in the fact that a linear equation, with no weights in any of its four variables, is being represented. Coincidence between Murray and his FIS appeared in 86% of the cases, while the rest were equally distributed at both sides of the central bar, supposing a histogram as the one shown in Figure 2.

## Discussion

In this study, the FL approach made it possible to measure knowledge, experience and intuition of a group of physicians to diagnose lung injury severity. It provides four main observations of interest. Firstly, the contiguous categories of the variables confirm the existence of fuzzy frontiers between them. Secondly, the existence of an overestimation in the interpretation of severity by the group surveyed. Thirdly, the origin of this overestimation was mainly due to different weights assigned to PO_2_/FiO_2 _and chest film variables. Finally, the FL methodology was able to overcome a series of restrictions that current tests have due to cut-offs.

As far as we know, two studies [7,8] have proposed to apply the FL theory in order to determine the severity of ARDS, but there is a lack of information about ALI-ARDS scoring and its relationship with human intuition. Steltzer *et al *[8,10] developed a model based on the fuzzy set theory to evaluate patients with ARDS using extracorporeal lung assist therapy through 25 different parameters observed at four points in time. They concluded that the fuzzy set theory could be useful to evaluate these patients under a controlled ARDS therapy. Velasevic *et al *[7] also developed a FIS to determine the degree of severity of pulmonary injury but certain differences with our work must be underscored. They introduced five other variables subdivided into five categories. The **c**ategories of four of them are fuzzy and determined *ad hoc*. Neither of them included a survey among physicians.

Our idea was to shape FL to the natural categorization into three zones, as our everyday vocabulary does. Murray's test breaks down each variable into five segments to reach a total of 625 combinations from which certain impossible combinations should be discarded. Leaving aside the fourth variable (chest film), which was considered just like in Murray's work, in the FIS we have only three regions per variable. But, the overlapping of membership functions results in three "pure" regions and another two or more corresponding to a superposition of curves (figure 1). All these regions have varied extensions as compared with the uniform and symmetric ones defined by Murray. Then, the fact of having set only three categories for each variable is not a restriction. Any intent to create more divisions is due to the pretension of having a certain systematization, as Murray does, but this intent turns out to be confusing at the intuitive level if at the time of alluding to one of them not all physicians are thinking about the same category. On the other hand, it is worth asking if similar results would have been obtained had we partitioned into just two regions. However, it could be anticipated that the physicians' intuition would naturally resist this oversimplification (for instance, splitting PO_2_/FiO_2 _ratio into normal and low would lead us to lose clinical precision). As far as the output is concerned, we decided to split Murray's mild to moderate band into two parts on the basis of another study [11]. We decided to use this cut-off score to better assess the transition between mild and moderate bands.

The points fitting process for membership functions gave us certain freedom as to the election of the most appropriate one available to us from the MATLAB repertoire. Eight of them were used for all variables. The fourth variable, the chest film, was not an issue in this work as it was considered that film inspection could only provide a 0, 1, 2, 3 or 4 quantized conclusion about the consolidated quadrants. This is an important restriction that the Murray test imposes as it does not allow for views that are not well-defined. The plasticity of the continuum makes it possible to deal with intermediate situations that a clinical eye could decide are worthy to be considered. Anyhow, this was not the case in this research work as the second questionnaire was extremely precise considering that only 0, 1, 2, 3 or 4 were the possible values for the consolidated quadrants.

The group surveyed showed a tendency to overestimation in the interpretation of severity (Figure 2). This fact is founded on two reasons. Low values of PO_2_/FiO_2 _and high values of consolidations were most important to diagnose severity. More so, better values of compliance and PEEP were not great determinants of amelioration. Through the FL methodology we can infer that our physicians are likely influenced in their diagnosis by the AECCC criterion which stresses the importance of PaO_2_/FiO_2_. These discrepancies evidenced that physicians' experience and intuition follow nonlinear paths, in opposition to the linearity of Murray's equation.

With the intention of unraveling the hidden membership functions that would lie within the Murray equation if it were interpreted as an equivalent FIS, we proceeded to fuzzify the Murray equation to obtain the membership functions to which Murray's reasoning would respond if we had surveyed him just as we did in the survey. We found a striking symmetry of the first three variables which is grounded on the linearity of the defined equation and the absence of different weights in its addends (Figure 3).

No matter which method of scoring is the most appropriate, all of them deal with an insurmountable hindrance: the need to fix a crisp number to pivot as a frontier between two categories of a variable (for instance, moderate and severe in AECCC), or to assign a unique representative number to an interval of values of a variable to be later included in an equation (Murray score). Even though a group of remarkable experts could agree on the variables to be considered as indispensable to characterize the degree of injury, it is almost impossible to fully agree on those crisp numbers that must define the frontiers as discussed before. Perhaps, sharp frontiers should not be used but rather replaced by fuzzy ones.

Intuition is an abstract entity that does not necessarily have to be transcribed in a linear manner, and it is clear that nonlinearity is omnipresent in the processes of Nature. A researcher might be tempted to simplify a problem too much, especially when many variables are involved. FL comes to solve this problem replacing causality with inference. In this way, the knowledge and experience of several specialists could be shaped into a FIS representing the know-how of the group.

This paper has some aspects that ought to be pointed out. The survey was conducted among a group of twelve physicians from an academic institution. A survey, as a sample of a larger population, entails an intrinsic uncertainty. Our survey is not a sample but the population itself. Thus uncertainty is not an issue. The group surveyed represents our intensive care team. With regard to overestimation and the different relevance of the variables this is not necessarily a generalizable result as other medical centres or critical care therapists would perhaps convey different criteria, but it serves the purpose of evincing what could be done if it were decided to survey a group of selected experts on the subject, which would not need to seek consensus now on crisp frontiers.

## Conclusions

1) The FL approach made it possible to measure knowledge, experience and intuition as they appear in physicians' thinking. FL methodology could overcome a series of restrictions that current tests have due to cut-offs.

2) Fuzzy frontiers and different diagnoses provided by physicians, which are usually an impediment to reach consensus, have now been integrated to shape the criterion of the group as a whole.

## Abbreviations

ICU: Intensive Care Unit; ARDS: Acute Respiratory Distress Symptom; ALI: Acute Lung Injury; AECCC: American-European Consensus Conference Committee; FL: Fuzzy Logic; FIS: Fuzzy Inference System

## Competing interests

The authors declare that they have no competing interests.

## Authors' contributions

CED conceived the study, and participated in its design, development, statistical analysis and interpretation, and drafted the manuscript. ELD participated in the design, coordination and interpretation, and helped to draft the manuscript. Both authors have read and approved the final manuscript.

## Appendix I Brief Introduction to Fuzzy Logic

We can synthesize the whole process of making up a FIS in the following four steps:

1. Fuzzification of input

2. Application of operators

3. Application of implication

4. Defuzzification

In the known theory of sets an element pertains or does not pertain to a set (1 or 0, respectively). In a fuzzy set its frontier is not so well defined, thus surrendering a membership value between 0 and 1. The concept of membership degree is subjective and dependent on domain.

Once all functions associated to fuzzy concepts (*membership functions*) have been defined, we face the issue of how to interpret the logical operators "AND", "OR", "NOT" and the one of implication "IF...THEN" that relates them.

When dealing with classical logic (binary logic) we have only two truth values, 0 (false) and 1 (true).

As we said before, our new logic permits any value between 0 and 1; then, a new operator is needed that if led to the limit of classical logic retakes the old values of the latter. For "AND" such a *possible *one could be the 'minimum function' min(A,B) which provides the minimum value of A and B. Following the same reasoning, we could associate "OR" with the 'maximum function' and NOT A with 1-A.

**Figure 4:**
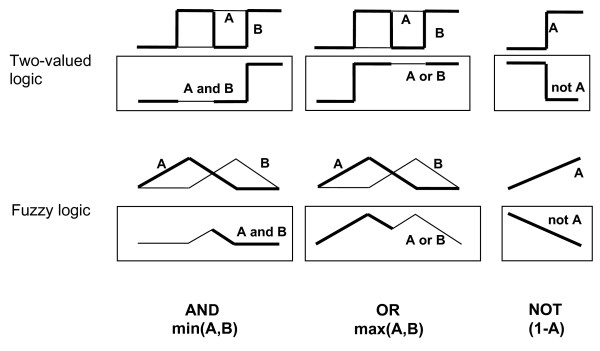
**Classical and fuzzy operators**. The application of classical operators is restricted to only two-valued functions whereas generalized ones can also be applied to continuous functions.

As an example, in Figure 4 we can see an application to step-like functions A and B for classical logic, and triangle-like functions A and B for fuzzy logic, rendering a multi-valued output in this case.

A fuzzy if-then *rule *assumes the form

If x is A then y is B

In general, A or B are linguistic values represented by fuzzy sets on the ranges (universes of discourse) X and Y, respectively. For example, X can be PaO_2_/FiO_2_, A high, Y diagnosis and B no-injury; x and y will be particular values of the corresponding universes of discourse. Thus, we could have the following statement:

If PaO_2_/FiO_2 _= 225 is high then diagnosis is no-injury

This diagnosis (consequent or conclusion) has a certain *degree *of truth (between 0 and 1) which will be determined by the *degree *of truth of the proposition before 'then' (antecedent). The universe of discourse of the diagnosis could be a discrete or a continuous one. The latter means that our output will not be a number but a membership function like the ones in the input (Mamdani type FIS) and, the former, just a crisp value (Sugeno type FIS). The latter case is the one we implemented in this article, where diagnosis was assigned only four categories (crisp values) of severity (no-injury, mild, moderate and ARDS).

If-then rules do not present much difficulty in classical sets: if the premise is true then the conclusion is true; if the premise is false then the conclusion is false (to be understood as the consequent not occurring or its action not executed). If we relax the restrictions of two-valued logic and allow the antecedent to be a fuzzy statement, then we should propagate its same degree of certainty to our consequent whether a crisp number (Sugeno) or a membership function (Mamdani). In the latter case, this implies chopping off the top of the function at the level of that certainty. The process just described can be represented by min(A,B) that returns our B function truncated at the height at which A membership is.

A FIS with only one rule does not do much good, as its potentialities cannot be deployed. Our FIS has not one but a collection of rules, and the interplay of all of them will determine a final and unique conclusion. This is known as *aggregation*. What comes next is a process of *defuzzification *to obtain a final crisp number representing the output of the several crisp values at the input. If conclusions were membership functions, then aggregation makes sense and a kind of OR operator among them is to be applied. However, if we have only crisp numbers (Sugeno), neither of the two (*aggregation *or *defuzzification*) would be necessary, except for the presence of more than one crisp number for only one input, as is our case. In the latter situation a criterion must be defined to reduce such vagueness to only one crisp number. For this last step we applied a weighted sum. It must be noticed that this sum will generally render a fractional number which will be rounded to 0, 1, 2 or 3 to associate it with one of the four possible severity conditions.

By no means are these elections of fuzzy operators the only possible ones, though all of them must contain the classical limits and satisfy certain mathematical properties [6,12]. For example, for the AND operator we have adopted the 'product function' prod(A,B) = a * b and for the OR operator, the 'probabilistic OR' method probor(A,B) = a + b - a * b, a and b being the truth values. Choosing one or another has to do with our decision to be more restrictive or permissive with our truth values. Custom-tailored operators are also feasible.

## Appendix II

### a) Building a membership function

Given a variable, the values in the corresponding questionnaire were represented in the abscissa, whereas the ordinate was the density of the number of observations. So, for example, if PaO_2_/FiO_2 _is 150 and 7 out of 12 physicians considered it to be a severe value, then the respective density of membership of that value to the category of severity is 7/12 = 0.58. As there was a finite number of values in the questionnaires, an interpolation was necessary. To accomplish this, we had to choose from a collection of analytical curves to fit the points in the most accurate way possible (MATLAB Software, The MathWorks, Inc.). Our discrete set gives rise to a continuous domain, thus letting us get a membership value for "any" value of the variable (see all of them in Figure 1).

### b) Chest film membership function

Translated to fuzzy domain we decided its membership function (Figure 1d) should be a generalized bell-shaped one. Notice that even in this case we have no defined frontiers as we transit from a 0 value (no consolidation) to a 1 value (one quadrant consolidation), or from 1 to 2, or 2 to 3, or 3 to 4. So, for example, in a small neighbourhood of the intermediate point 1.5 between 1 and 2 we would assign a greater than zero membership value to two functions.

### c) Attainment of the FIS output

The fourth questionnaire represents the building of the rules that connect the input of linguistic variables associated with physiological parameters to a precise diagnosis of severity, as follows:

If PaO_2_/FiO_2 _is **normal**, and Compliance is **normal**, and PEEP is **moderate**, and Rx is **one consolidation**, then injury is **absent**.

Or,

If PaO_2_/FiO_2 _is **normal**, and Compliance is **normal**, and PEEP is **moderate**, and Rx is **one consolidation**, then injury is **mild**.

Or,

If PaO_2_/FiO_2 _is **normal**, and Compliance is **normal**, and PEEP is **moderate**, and Rx is **one consolidation**, then injury is **moderate**.

This example refers to a case where the same linguistic input has originated three rules, giving rise to three crisp outputs, each one with a certain weight. Here again we face another expression of fuzziness as one, two and even three choices appeared on certain lines after collecting all the answers of the survey. Thus, not only the categories but also the diagnoses of injury are fuzzy. More than one answer per line gave rise to the same number of rules, each one weighted by a coefficient resulting from dividing the number of times a cell was chosen by twelve (total number of those surveyed). The obtained FIS resulted in a collection of 222 rules. At this point we were in a position to feed our FIS with any possible values of the four input variables and obtain a collection of all possible weighted outputs of the rules. The last step is turning that fuzziness into a crisp number, the *degree of severity*, obtained as the weighted mean of that collection. This mean value pertains to a continuum that embraces the [0 to 3] interval. As the level of injury has to be one among four possible (0, 1, 2 or 3), we made the decision of choosing that one which is the nearest to the value obtained.

### d) Choice of data procedure

Data were chosen in the following manner: a) the central value of each interval defined by the Murray test; b) two close values surrounding the frontiers between intervals; and c) for chest film, values from 0 to 4, according to Murray's definition. This selection resulted in 18 values for each one of the first two variables, 13 for the third and 5 for the last. This totaled 21,060 quadruplets in the form of a large four-column matrix, where some of its rows could be:

250   55   8     1

180   50   12   2

and where each column represents selected values of PaO_2_/FiO_2_, compliance, PEEP and No. of consolidations, respectively. Every row gives rise to a diagnosis from the survey and from Murray. Certain extreme combinations, that could never have appeared, as for example, those with PaO_2_/FiO_2 _= 450, and chest film = 4 or PaO_2_/FiO_2 _≤ 137, and chest film 0 or 1, were dismissed. This depuration, which left us with number 18,013, is in accordance with the proposed combinations in the fourth questionnaire, where extreme ones had not been contemplated either.

### e) Management of the output of both methods

The output of the FIS for each quadruplet of input values was a fractional number that was rounded off to its nearest integer 0, 1, 2 or 3, as stated in the Method section. This is a vector that we will call S. Quadruplets were also evaluated according to the Murray test and results were assigned values 0, 1, 2 or 3 depending on whether they had fallen in intervals [0, 0.1], (0.1, 1.5], (1.5, 2.5] or (2.5, ∞), respectively. This other vector will be called M. Subtracting S-M resulted in a vector (length 18,013) with -2, -1, 0, 1, 2 as possible values.

### f) Different weight assigned to variables between the surveyed group and the Murray score

**Figure 5:**
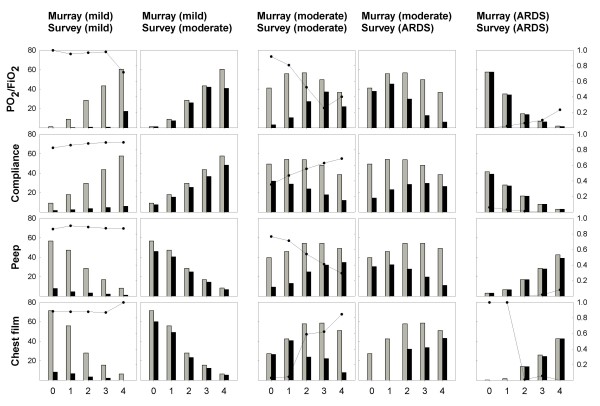
**Comparison between Murray's and the Survey's diagnostics for all variables according to severity**.

Figure 5 below shows the comparison between Murray and Survey diagnosing according to severity (mild, moderate, ARDS) for all variables. The three blocks of column graphs represent Murray's severity degrees. All graphs are pairs of histograms referred to Murray (grey bars) and the survey (black bars). Abscissas correspond to the Murray intervals of the different variables (including 0 to 4 consolidations for the last one). Bars represent the percentage distribution (left scale) amongst the whole amount of data (18,013 cases) per Murray band of cases needed to match a certain degree of severity (according to Murray or to the Survey) within the universe of cases, with that same severity, determined by Murray. Murray's histograms repeat themselves in both columns of each one of the first two blocks as they address the same severity. Those of the survey correspond to the same level as Murray's on the left column and one more on the right column of each block. The curves indicate proportion of discrepancy (right scale) with respect to Murray for each one of his bands.

Had there been a total diagnostic coincidence between Murray and the survey, the figure should contain three blocks but with one column each, its histograms should look like totally coincident pairs of bars and the proportion of discrepancy curve should be a flat one at the bottom of the graphs. Instead, the figure shows that as severity decreases, from right to left, a progressive decoupling of diagnosis between Murray and the survey is apparent. From almost total coincidence to almost total discrepancy, the intermediate state of the degree of severity serves the purpose of clearly showing how discrepancies originated.

For the cases considered as moderate by Murray, the main cause of overestimation of injury in the survey was the assignment of greater importance to low values of PaO_2_/FiO_2 _and high values of consolidations, as can be seen in the first column of the block if we search for the region towards which the proportion of the discrepancy curve climbs. Considering only these regions and carrying out proportion tests between pairs of variables, we found that all the higher values of the discrepancy curve are significant (p < 0.0001). The decreasing bar heights of the survey in these regions corresponds to the migration of cases towards the contiguous right column of one more degree of severity. Based on this rationale, we can see that cases with better values of Compliance and PEEP also migrate in the same direction. The apparent paradox of Compliance and PEEP behavior must be preferably interpreted as though Murray were ameliorating the diagnosis faster than the survey does for these better values. The higher values assumed by the discrepancy curves are found in PaO_2_/FiO_2 _and chest film variables. It must also be noted when looking at the bottom histograms in the survey's ARDS columns that there is no possibility for them to assign that severity in cases of zero or one consolidation, regardless of the values of the other variables. Summarizing, they neither decrease the importance of low values of PaO_2_/FiO_2 _or high values of consolidations as quickly as Murray does, nor do they emphasize better values of compliance and PEEP as quickly as Murray does. At mild severity, virtually the whole range of variables turns out to be overestimated while, as it was said before, maximal coincidence was found for ARDS, where overestimation was impossible.

## Pre-publication history

The pre-publication history for this paper can be accessed here:

http://www.biomedcentral.com/1472-6947/10/70/prepub
